# Laser Cladding for Diamond-Reinforced Composites with Low-Melting-Point Transition Layer

**DOI:** 10.3390/ma18102402

**Published:** 2025-05-21

**Authors:** Yongqian Chen, Yifei Du, Jialin Liu, Shanghua Zhang, Tianjian Wang, Shirui Guo, Yinghao Cui, Xiaolei Li, Bo Zheng, Yue Zhao, Lujun Cui

**Affiliations:** 1 School of Mechatronics Engineering, Zhongyuan University of Technology, Zhengzhou 451191, China; katila02@163.com (Y.D.);; 2 Zhengzhou Key Laboratory of Laser Additive Manufacturing Technology, Zhengzhou 451191, China; 3 Henan Key Laboratory of General Aviation Technology, Zhengzhou University of Aeronautics, Zhengzhou 450046, China; 4 Institute of Manufacturing Engineering, Huaqiao University, 668#, Xiamen 361021, China

**Keywords:** laser cladding, graphitization, diffusion barrier, diamond tools, laser processing

## Abstract

To address the graphitization of diamond induced by high temperatures during laser cladding of diamond-reinforced composites, this study proposes a laser cladding method utilizing Inconel 718 (IN718) nickel-based alloy as a transition layer which has a lower melting point than the substrate of 45# steel. And then, in order to analyze the detailed characteristics of the samples, scanning electron microscopy (SEM), EDS, Raman spectral analyzer, super-depth-of-field microscope, and friction tests were used. Experimental study and the test results demonstrate that the IN718 transition layer enhances coating performance through dual mechanisms: firstly, its relatively low melting point (1392 °C) reduces the molten pool’s peak temperature, effectively suppressing thermal-induced graphitization of the diamond; on the other hand, simultaneously it acts as a diffusion barrier to inhibit Fe migration from the substrate and weaken Fe–C interfacial catalytic reactions. Microstructural analysis reveals improved diamond encapsulation and reduced interfacial sintering defects in coatings with the transition layer. Tribological tests confirm that samples with the transition layer L exhibit lower friction coefficients and significantly enhanced wear resistance compared to those without. This study elucidates the synergistic mechanism of the transition layer in thermal management optimization and interfacial reaction suppression, providing an innovative solution to overcome the high-temperature damage bottleneck in laser-clad diamond tools.

## 1. Introduction

Diamond material is widely used in high-precision machining due to its high hardness and high thermal conductivity [1,2]. Diamond tools combine diamond particles with metal powder, resin, or vitrified bond to form cutting edge or grinding layer by electroplating, hot pressing sintering, chemical vapor deposition, or brazing process. At present, diamond grinding wheels, diamond drills and other processing tools are widely used in high-tech fields such as building materials, precision manufacturing, electronic information, and geological exploration. Its efficient processing performance and longer service life significantly improve the efficiency and economy of industrial production [3,4].

Laser cladding diamond technology forms metallurgically bonded coatings on diamond surfaces through high-energy interactions between the laser and substrate materials. This method offers advantages such as a small heat-affected zone, high interfacial bonding strength, low material dilution rate, and high forming precision. In recent years, laser cladding diamond technology has gained attention as an advanced tool preparation method that combines efficient forming and functional customization. However, challenges in synergistically regulating thermodynamic constraints and interfacial reaction-diffusion mechanisms remain critical bottlenecks for performance breakthroughs. In 2012, M. Iravani et al. optimized the laser cladding process for copper-tin-titanium-diamond composite coatings, demonstrating that titanium enhances bonding strength by in situ forming a TiC interfacial layer. Pulsed lasers were shown to suppress diamond graphitization and control TiC layer thickness [5]. Building on this, Pang A. et al. addressed interfacial bonding issues by employing Cr-coated diamond particles and Ni-Cr-B-Si alloy composites, achieving metallurgical bonding through a “diamond-chromium carbide-metal chromium” core-shell structure. This improved coating wear resistance by 4.6 times, confirming that surface metallization effectively mitigates high-temperature diamond damage [6]. However, an interfacial reaction study by Rommel et al. found that even when protected by metal plating, the laser heat source led to the formation of an 8 μm graphitized transition layer on the diamond surface, a phenomenon that enhances the mechanical embedding but weakens the chemical bonding, revealing the decisive influence of the process of heat management on the interfacial integrity [7].

Although process optimization and interface modification have significantly enhanced the metallurgical bonding strength between diamond and matrix, thermal-induced diamond graphitization remains the core issue limiting tool performance degradation [8]. In 2023, Ma et al. elucidated a unique bimodal temperature evolution mechanism in molten pools through three-dimensional transient heat transfer modeling. They identified dual temperature peaks: the primary peak (1491.6 °C) originating from molten pool contact heat transfer, and the secondary peak (1896.1 °C) resulting from thermal accumulation, establishing quantitative relationships between process parameters, temperature profiles, and graphitization degrees [9]. Chen et al. further demonstrated via multiphysics-coupled simulations that laser path offset modulates bimodal structural morphology. When offset exceeds 25 μm, bimodal fusion generates single-peak temperature curves, with particle size reduction to 45–53 μm elevating diamond peak temperatures by approximately 150 °C compared to 74–90 μm particles, revealing the amplification effect of particle size on thermal exposure [10]. These numerical studies quantitatively deciphered diamond’s transient heat transfer behavior under extreme thermal cycling, providing critical theoretical support for determining the graphitization temperature threshold (1491.6 °C) and interfacial reaction kinetics. In 2022, G Zhong et al. discovered through cutting force modeling that natural Cape Ia diamonds with specific crystallographic orientations reduce tool stress concentrations and suppress graphitization wear [11]. Concurrently, F Wu et al. demonstrated that micro-texture engineering of diamond coatings delays graphitization kinetics (35% reduction in graphitization degree), albeit at the expense of surface integrity (Ra roughness increased by 0.8 μm) [12].

To address the aforementioned challenges, this study proposes an innovative process strategy: Pre-depositing an In718 nickel-based alloy transition layer on 45 steel substrates, followed by laser cladding to fabricate diamond-reinforced metal matrix composites [10]. The low melting point of In718 effectively reduces the peak temperature of the molten pool, thereby suppressing diamond graphitization, while its low laser absorptivity minimizes thermal input to mitigate thermal damage. Simultaneously, the transition layer acts as a diffusion barrier to block Fe migration from the substrate to the clad layer, weakening Fe–C interfacial catalytic effects and preserving diamond structural stability. This work systematically investigates the influence mechanisms of the In718 transition layer on cladding morphology, diamond encapsulation efficiency, graphitization degree, and tribological performance, elucidating the dual role of low-melting-point transition layers in thermodynamic regulation and interface optimization. The findings provide novel solutions for addressing high-temperature damage and interfacial failure in laser-clad diamond composites, while establishing theoretical and technical foundations for industrial-scale production of ultra-wear-resistant coatings.

## 2. Experimental Materials and Equipment

### 2.1. Experimental Materials

**Figure 1:**
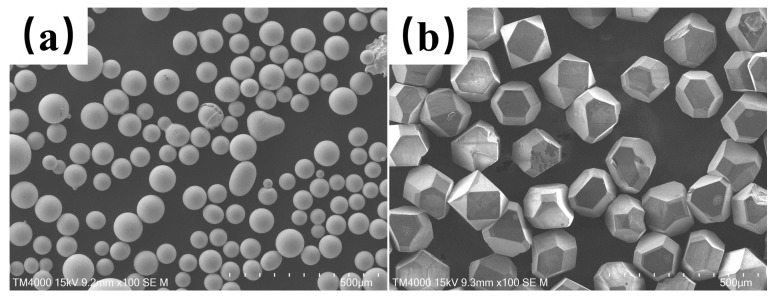
In718 alloy powder as well as diamond particles microscopic morphology; (**a**) In718 alloy powder, (**b**) diamond particles.

In this experiment, gas-atomized In718 alloy powder (supplied by Zhejiang Yatong Welding Materials Co., Ltd., Hangzhou, China) and diamond particles (manufactured by Huanghe Whirlwind Co., Ltd., Xuchang, China) were used as raw materials. The In718 alloy powder had a particle size range of 150–270 mesh, with specific composition details listed in Table 1. The diamond particles were 100 mesh in size and uncoated. This diamond particle size was selected to prevent thermal ablation or excessive graphitization during laser cladding experiments, phenomena typically observed with undersized diamond particles. The morphologies of the metal powder and diamond particles are shown in Figure 1. Prior to experimentation, the In718 alloy powder and diamond particles were uniformly mixed at a volume ratio of 80:20 (80% In718, 20% diamond) using a planetary ball mill (QM-3SP2 planetary ball mill, Nanjing, China) for 2 h. The mixed powder was then dried in an oven (HD-E804-45A Blower Drying Oven, Hangzhou, China) at 120 °C for 24 h to ensure moisture removal [13].

### 2.2. Experimental Setup

The cladding experiments were conducted using the iLAM^®^25Fpt-600 flexible robotic laser cladding system manufactured by Nanjing HuiRui Optoelectronics Technology Co., Ltd. (Nanjing, China). This system is equipped with a continuous fiber laser delivering a maximum output power of 6000 W and operating at a wavelength of 1064 nm. A circular adjustable laser spot was employed, with a fixed diameter of 3 mm in this study. The laser cladding system adopts a coaxial annular powder feeding nozzle to ensure powder uniformity and high utilization efficiency. A nitrogen gas (nitrogen purity 99.99%) cylinder station was integrated to create a protective atmosphere and supply carrier gas flow, minimizing oxidation risks for diamonds during processing. The 9MP powder feeder (Shanghai Meiteke Thermal Spray Co., Ltd., Shanghai, China) enabled pre-stirring and preheating operations, further enhancing powder feeding uniformity and utilization rate. Carrier gas-assisted powder feeding technology was utilized for long-distance powder delivery, with the carrier gas flow rate set at 9 L/min.

### 2.3. Experimental Program

**Figure 2:**
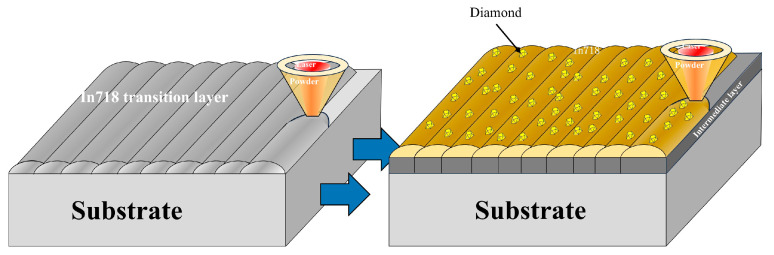
Schematic diagram of the preparation process of sample laser melting [12].

In this experiment, a 45 steel substrate with dimensions of 200 mm × 50 mm × 10 mm was used. The specific composition of 45 steel is shown in Table 2. A transition layer was pre-deposited on the substrate, followed by laser cladding of diamond-reinforced metal matrix composites; the melting point of Inconel is lower than that of steel 45, so in the manuscript we will refer to this layer as the low-temperature layer (LTL), as illustrated in Figure 2.

Prior to transition-layer preparation, the 45 steel substrate underwent surface grinding and polishing using an angle grinder (DELIXI H3 power tools, Wenzhou, China) to remove rust, oil stains, and surface contaminants. Inconel 718 alloy powder was then loaded into the powder feeder to fabricate the LTL, with process parameters listed in Table 3.

**Figure 3:**
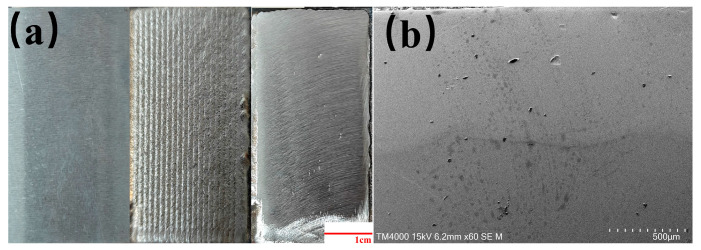
Shows the super-depth-of-field microscope and SEM images of the substrate and LTL surfaces. (**a**) Super-depth-of-field microscope images: from left to right are substrate, original LTL morphology, and polished LTL morphology; (**b**) SEM image showing the cross-sectional morphology of the LTL.

After deposition, the LTL was polished to a thickness of 1 mm, and its surface morphology is shown in Figure 3.

Both the polished 45 steel substrate (without an LTL) and the substrate with the LTL were mounted on the cladding system. Laser parameters included a power range of 600–1000 W (in 100 W increments), powder feeding rate of 29 g/min, scanning speed of 10 mm/s, and overlap rate of 40%. A total of 10 single-track cladding layers were deposited on each substrate, followed by 10 overlapping passes [14].

After coating preparation, the samples were sectioned into 20 mm × 20 mm × 10 mm dimensions using wire electrical discharge machining for characterization (Suzhou New Spark M332S, Suzhou, China). The sectioned samples were ultrasonically cleaned in an ultrasonic cleaner, air-dried, and then hot-mounted using a metallographic mounting press (Shanghai Minxin Instrument Testing Co., Ltd.) at 170 °C for 10–20 min. Following mounting, the specimens were pre-ground using 400 to 800 grit SiC abrasive papers on an MPZ-1 metallographic grinding/polishing machine (Shanghai Minxin Instrument Testing Co., Ltd., Shanghai, China) following the ascending order of grit size. After pre-grinding, the polishing cloth was replaced, and a 3.5 μm water-soluble diamond polishing compound was applied as the abrasive for final polishing. The polished samples were re-cleaned in an ultrasonic cleaner for 1 h to remove residual polishing agents, wiped with an alcohol-soaked lint-free cloth, and air-dried for subsequent experimental analysis [15].

### 2.4. Testing Equipment

A TM4000plus (Hitachi, Ltd., Tokyo, Japan) scanning electron microscope (SEM) was used to characterize the particle morphology of In718 alloy powders and diamond micro powders, as well as the surface profile morphology of the samples. The SEM system was equipped with a Quantax 75 Energy Dispersive X-ray Spectroscopy (EDS) system for elemental composition analysis of scanned regions (Berlin, Germany). A Horiba Xplore Plus laser confocal Raman microscope was employed to analyze the graphitization of diamond in the composites. The system utilized a 532 nm green laser source with a power of 50 mW, a laser spot diameter of 2 μm, and a spectral range of 500 cm^−1^ to 2000 cm^−1^. The friction and wear tests were conducted using a controlled-atmosphere micro tribometer (WTM-2E, Lanzhou Zhongke Kaihua Technology Development Co., Ltd., Lanzhou, China) with stainless steel balls as counterface materials. The test parameters included a normal load of 200 g, sliding duration of 1 h, motor speed of 300 rad/min, and rotation radius of 2 mm. The sample morphology was characterized using the Easy Zoom5 super-depth-of-field microscope optical microscope from Motic Instruments Group Co., Ltd. (Xiamen, China). This microscope features ultra-high true-color reproduction capability and is equipped with a high-brightness LED light source with adjustable intensity, enabling high-quality image acquisition under variable illumination conditions to clearly reveal the sample’s microscopic morphology and surface characteristics.

## 3. Results and Discussion

### 3.1. Analysis of Sample Morphology

#### 3.1.1. Comparison of Cladding Morphology with and Without Transition Layer

**Figure 4:**
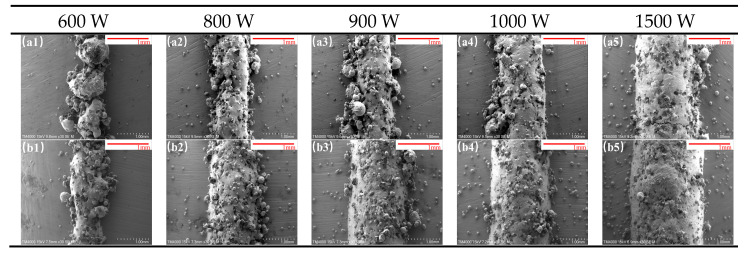
SEM characterization of single-track clad layers under laser powers of 600–1500 W. Subfigures (**a1**–**a5**) and (**b1**–**b5**) represent samples without and with LTLs, respectively.

Figure 4(a1–a5,b1–b5) illustrate the cladding results under laser powers ranging from 600 W to 1500 W. Figure 4(a1–a5,b1–b5) correspond to single-track cladding results without and with an LTL, respectively, under a fixed scanning speed of 10 mm/s and powder feeding rate of 29 g/min.

Comparative analysis reveals that at laser powers below 800 W, both samples exhibit morphological defects. However, the cladding layers with an LTL demonstrate superior surface integrity compared to those without. When the laser power exceeds 800 W, the samples with an LTL achieve well-formed cladding morphology, indicating effective bonding between the substrate and powder material. In contrast, for samples without an LTL, cladding layers formed at 800–1000 W exhibit poor morphology characterized by agglomerated metallic powder structures. No significant differences in cladding width or macroscopic morphology are observed between the two groups at laser powers above 1000 W. Judging solely by clad layer morphology, the 1500 W laser power yielded superior sample morphology; however, subsequent experimental characterization revealed that excessive laser power induced severe diamond sintering and complete thermal ablation in the clad layer. Consequently, subsequent experiments focused on moderate laser power samples (600–1000 W) as the primary characterization subjects.

At 900 W (Figure 4(a3,b3)), the sample without an LTL (Figure 4(a3)) displays incomplete single-track cladding, indicating insufficient bonding between the substrate and powder. In contrast, the sample with an LTL (Figure 4(b3)) demonstrates intact cladding morphology and effective substrate-powder bonding. This disparity arises because the In718 LTL has a lower melting point than the 45 steel substrate, enabling enhanced bonding with the powder material at reduced laser power.

The experimental results confirm that the LTL facilitates metallurgical bonding between the powder and substrate at lower laser powers. Reduced laser power lowers the molten pool temperature, thereby mitigating the risk of diamond graphitization [16].

#### 3.1.2. Comparison of Diamond Microstructure and Encapsulation Efficiency with/Without Transition Layer

**Figure 5:**
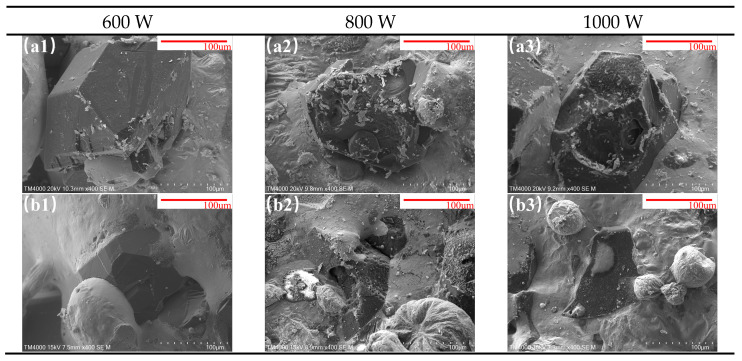
SEM characterization of diamond in cladding under laser powers of 600 W, 800 W, 1000 W. Subfigures (**a1**–**a3**) and (**b1**–**b3**) represent samples without and with LTLs, respectively.

Figure 5(a1–a3,b1–b3) present the cladding morphology under laser powers of 600 W, 800 W, and 1000 W. Figure 5(a1–a3,b1–b3) correspond to multi-track diamond-reinforced cladding results without and with an LTL, respectively, under a scanning speed of 10 mm/s and powder feeding rate of 29 g/min.

For samples without an LTL, the In718 matrix alloy exhibits minimal encapsulation of diamond particles at 600 W, with partial encapsulation only observed at 1000 W. In contrast, samples with an LTL achieve near-complete diamond encapsulation at 600 W, with further improvement at 800 W.

The results demonstrate that the LTL enhances the wetting behavior of the matrix alloy toward diamond. The lower melting point of the In718 LTL (1392 °C) compared to the 45 steel substrate (1500 °C) enables more thorough melting of the clad surface under identical laser power, thereby improving diamond encapsulation efficiency [17].

### 3.2. Diamond Graphitization

#### 3.2.1. Comparative Analysis of Raman Spectroscopy Data

**Figure 6:**
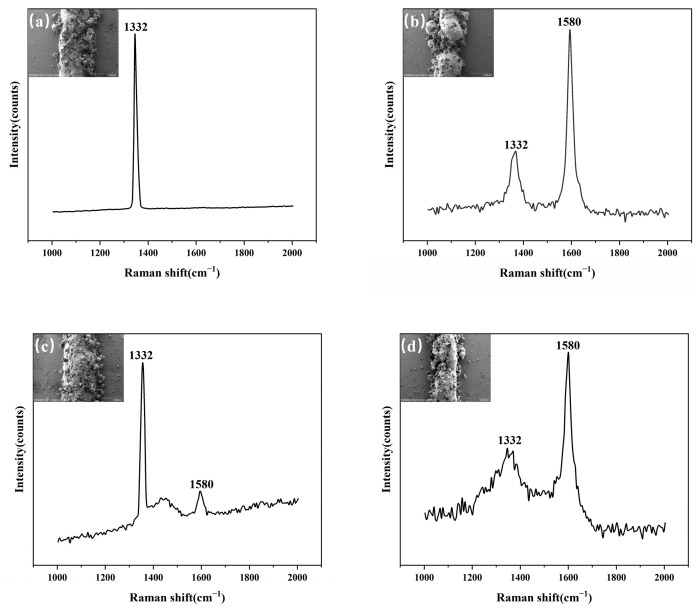
Raman spectra of diamond under varying laser powers: (**a**) 600 W with In718 LTL; (**b**) 600 W without LTL; (**c**) 800 W with In718 LTL; (**d**) 800 W without LTL.

Figure 6 shows the Raman spectra of samples with and without an LTL under laser powers of 600 W and 800 W (scanning speed: 10 mm/s; powder feeding rate: 29 g/min), with a spectral range of 1000 cm^−1^–2000 cm^−1^.

Figure 6a (with In718 LTL at 600 W laser power) exhibits a sharp diamond characteristic peak at 1332 cm^−1^ in the Raman spectrum, with no extraneous peaks, indicating an intact diamond lattice and no graphitization. In contrast, the sample without an LTL (Figure 6b) shows a distinct graphitization peak at 1580 cm^−1^ alongside the diamond peak, with an intensity ratio ID/IG = 0.326 [9], confirming partial surface graphitization of the diamond. At 800 W laser power, the transition-layer sample (Figure 6c) displays enhanced intensity and sharper profile of the 1332 cm^−1^ peak compared to the 600 W group, with no graphitization peaks, suggesting improved diamond-matrix interfacial bonding without thermal damage. Conversely, the non-transition-layer sample (Figure 6d) exhibits significantly intensified graphitization peak intensity (ID/IG = 0.415) and a defect-induced D’ peak at 1350 cm^−1^ [18], indicating accelerated structural defects and phase transitions under high-temperature molten pool conditions. For transition-layer samples at 600 W and 800 W, the increased 1332 cm^−1^ peak intensity and narrowed peak width demonstrate that higher laser power enhances diamond–binder interfacial bonding without inducing thermal degradation. However, in non-transition-layer samples, the ID/IG ratio rises from 0.326 to 0.415 with increasing power (600 W → 800 W), accompanied by the emergence of the D’ peak, which reflects intensified graphitization due to elevated molten pool temperatures.

The In718 LTL (melting point: 1392 °C) reduces molten pool temperatures compared to the 45 steel substrate (1500 °C), effectively suppressing diamond graphitization below its critical threshold (850 °C in air) [19]. Additionally, the LTL lowers laser energy absorption, further minimizing thermal input to the molten pool. Within the 600–800 W range, the In718 LTL significantly reduces diamond graphitization. Transition-layer samples completely inhibit graphitization at both 600 W and 800 W, whereas non-transition-layer samples exhibit progressive graphitization with increasing power.

#### 3.2.2. Comparative Analysis of Diamond Sintering Degree

**Figure 7:**
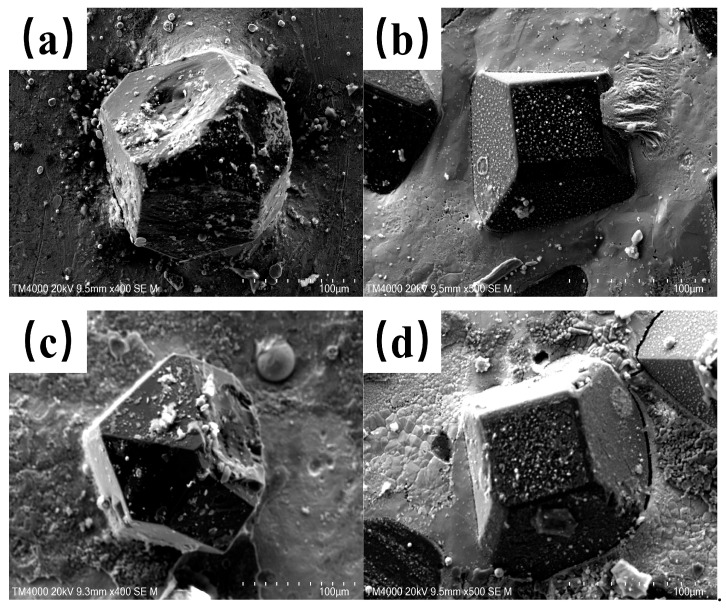
SEM micrographs of composite coatings: (**a**) 45 steel substrate at 600 W; (**b**) In718 LTL at 600 W; (**c**) 45 steel substrate at 800 W; (**d**) In718 LTL at 800 W.

Figure 7 presents the microstructural morphologies of polished cross-sections for samples without and with an LTL under laser powers of 600 W and 800 W (scanning speed: 10 mm/s; powder feeding rate: 29 g/min), observed via scanning electron microscopy (SEM).

Comparative analysis reveals that non-transition-layer samples exhibit pronounced diamond particle sintering, whereas transition-layer samples with In718 demonstrate significantly reduced sintering phenomena. Across both laser powers, transition-layer samples show milder sintering, while non-transition-layer samples exhibit progressively intensified sintering with increasing laser power [20].

This behavior stems from the lower melting point of In718 (1392 °C), which reduces molten pool temperatures during laser cladding. Lower molten pool temperatures suppress diamond sintering kinetics, as thermal exposure to diamond particles is minimized. Consequently, the In718 LTL enables laser cladding at reduced thermal inputs, effectively mitigating diamond sintering [21].

### 3.3. Comparative Analysis of Microstructural Elemental Variations

**Figure 8:**
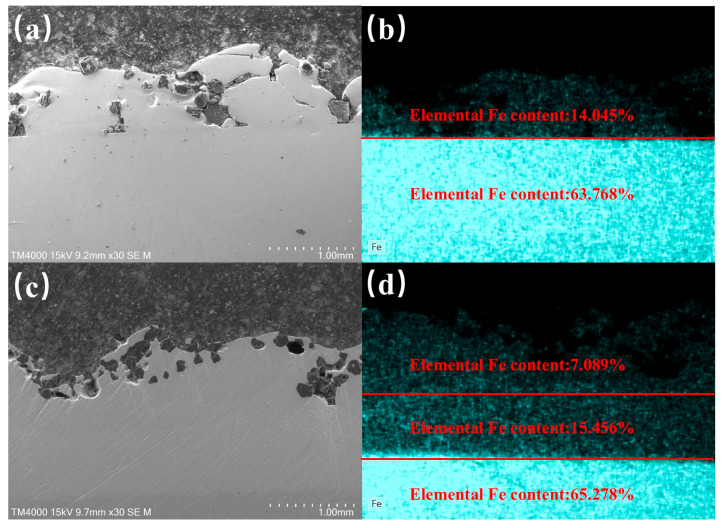
SEM-EDS analysis of Fe distribution: (**a**) 45 steel substrate with composite cladding at 900 W; (**b**) Fe content in substrate and cladding layer; (**c**) In718 LTL with composite cladding at 900 W; (**d**) Fe content in substrate, LTL, and cladding layer.

Figure 8 shows SEM-EDS results for multi-track cladding samples with and without an LTL, fabricated using In718 alloy powder mixed with diamond particles under laser power 900 W, scanning speed 10 mm/s, and powder feeding rate 29 g/min. EDS measurements of the samples were surface scans, and three surface scan measurements of the same sample were averaged.

In the EDS elemental mapping of Fe, the transition-layer sample exhibits a distinct intermediate layer between the 45 steel substrate and the diamond-In718 cladding layer, whereas the non-transition-layer sample shows direct contact between the cladding layer and the substrate. Quantitative EDS analysis reveals significant differences in Fe content: Non-transition-layer sample: Two layers with Fe concentrations of 63.768% (substrate) and 14.045% (cladding layer); Transition-layer sample: Three layers with progressively decreasing Fe concentrations: 65.278% (substrate), 15.456% (In718 LTL), and 7.089% (cladding layer).

These results demonstrate that the In718 LTL effectively suppresses Fe migration into the cladding layer [22]. Fe atoms, with unpaired electrons in their valence shells, exhibit high chemical reactivity that facilitates interactions with diamond lattice electrons. Such interactions promote covalent bond formation between Fe and carbon atoms, leading to structural reorganization of diamond. Specifically, Fe catalyzes the breakdown of tetrahedral diamond bonds and the rearrangement of carbon atoms into graphite-like layered structures [23]. The reduced Fe content in the cladding layer mitigates this catalytic effect, thereby inhibiting graphitization.

### 3.4. Comparative Analysis of Friction Performance

**Figure 9:**
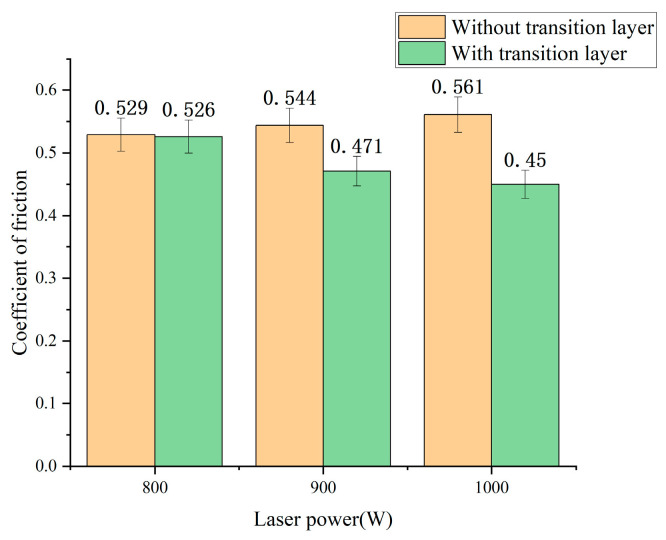
Average friction coefficients of samples without/with LTL at 800–1000 W laser power.

To investigate the influence of the LTL on the tribological properties of laser-clad diamond composites, the coefficient of friction tests were conducted on samples without and with LTLs under different laser power conditions (800 W, 900 W, and 1000 W). Results are shown in Figure 9.

Figure 9 demonstrates that under identical laser power conditions, transition-layer samples exhibit lower friction coefficients compared to non-transition-layer samples. For instance, at 900 W, the friction coefficient decreases from 0.544 (non-transition layer) to 0.471 (transition layer). At 1000 W, the friction coefficient further reduces from 0.561 (non-transition-layer) to 0.45 (transition layer). This improvement is attributed to the LTL’s ability to regulate molten pool temperature. As discussed in Section 3.2, the In718 LTL suppresses diamond graphitization and sintering by lowering molten pool temperatures, preserving diamond particle integrity and hardness. Additionally, the LTL enhances the encapsulation of diamonds by the binder (In718 alloy) (Section 3.1.2), reducing microdefects caused by un-melted powder agglomeration or poor interfacial bonding, thereby improving wear resistance [24].

For non-transition-layer samples, the friction coefficient initially increases and then decreases with rising laser power. This trend correlates with partial diamond graphitization (Section 3.2.1) and intensified Fe migration (Section 3.3) under high-power conditions. Graphitization reduces diamond hardness, while Fe presence may catalyze diamond-to-graphite phase transformation, forming soft phases that exacerbate adhesive wear [25]. In contrast, transition-layer samples show a continuous decline in friction coefficient with increasing power. The LTL not only inhibits graphitization but also minimizes thermal damage by reducing molten pool temperatures, achieving denser coating consolidation (Section 3.1.1) and more uniform diamond distribution. Furthermore, it blocks Fe diffusion into the cladding layer (Section 3.3), mitigating Fe-induced structural degradation of diamonds and significantly enhancing wear resistance [26].

## 4. Conclusions

This study successfully achieved molten pool temperature regulation and effective suppression of graphitization in laser-clad diamond composites by introducing an Inconel 718 (In718) Ni-based LTL. Experimental results demonstrate that the In718 LTL (melting point: 1392 °C) reduces peak molten pool temperatures due to its lower melting point and reduced laser energy absorption, significantly delaying diamond graphitization under high-temperature conditions and lowering sintering tendencies. Furthermore, the LTL inhibits Fe diffusion from the substrate, mitigating Fe–C interfacial catalytic effects and enhancing the structural stability of diamond. In friction and wear tests, transition-layer samples exhibited reduced friction coefficients compared to non-transition-layer samples at 1000 W laser power.

The innovation of this work lies in resolving the critical contradiction between thermal damage and interfacial failure in conventional laser-clad diamond technologies through coupled material design and process optimization, providing theoretical and technical pathways for industrial-scale fabrication of high-wear-resistance, long-service-life composite coatings. Future research may explore the potential of multi-component LTL materials (e.g., ceramic/metal gradient layers) to optimize the thermo-mechanical synergy of coatings and expand their engineering applications in extreme conditions, such as ultra-high-speed cutting and high-temperature corrosive environments.

## Figures and Tables

**Table 1:** Chemical composition of In718 alloy powder.

	Cr	Ni	Nb	Mo	Al	Ti	Si	Mn	Cu	O	Fe
Mass ratio	19.54	52.05	5.02	3.07	0.46	0.91	0.32	0.019	0.0017	0.0287	/

**Table 2:** Chemical composition of No. 45 steel.

	C	Si	Mn	S	P	Ni	Cr	Cu	Fe
Mass ratio	0.45	0.2	0.55	0.012	0.022	0.01	0.03	0.02	/

**Table 3:** Parameters of LTL preparation.

Process Parameters	Numerical Value
Laser power/W	1100
Scanning speed/mm/s	10
Powder feed rate g/min	29

## Data Availability

The original contributions presented in this study are included in the article. Further inquiries can be directed to the corresponding author.
